# The Protective Effect of DiDang Tang Against AlCl_3_-Induced Oxidative Stress and Apoptosis in PC12 Cells Through the Activation of SIRT1-Mediated Akt/Nrf2/HO-1 Pathway

**DOI:** 10.3389/fphar.2020.00466

**Published:** 2020-04-15

**Authors:** Jing Lu, Qingxia Huang, Dongmei Zhang, Tianye Lan, Ying Zhang, Xiaolei Tang, Peng Xu, Dexi Zhao, Deyu Cong, Daqing Zhao, Liwei Sun, Xiangyan Li, Jian Wang

**Affiliations:** ^1^Research Center of Traditional Chinese Medicine, the Affiliated Hospital of Changchun University of Chinese Medicine, Changchun, China; ^2^Jilin Provincial Key Laboratory of BioMacromolecules of Chinese Medicine, Changchun University of Chinese Medicine, Changchun, China; ^3^Key Laboratory of Active Substances and Biological Mechanisms of Ginseng Efficacy, Ministry of Education, Changchun, China; ^4^Scientific Research Office, the Affiliated Hospital of Changchun University of Chinese Medicine, Changchun, China; ^5^Department of Encephalopathy, the Affiliated Hospital of Changchun University of Chinese Medicine, Changchun, China; ^6^Jilin Ginseng Academy, Changchun University of Chinese Medicine, Changchun, China; ^7^Department of Tuina, the Affiliated Hospital of Changchun University of Chinese Medicine, Changchun, China

**Keywords:** DiDang Tang, neuroprotection, oxidative stress, apoptosis, aluminum

## Abstract

Aluminum (Al) is considered a pathological factor for various neurological and neurodegenerative diseases, such as Alzheimer’s disease (AD) and Parkinson’s disease (PD). The neurotoxicity of aluminum can cause oxidative brain damage, trigger apoptosis, and ultimately cause irreversible damage to neurons. DiDang Tang (DDT), a classic formula within traditional Chinese medicine for promoting blood circulation and removing blood stasis and collaterals, is widely used for the treatment of stroke and AD. In this study, models of oxidative stress and apoptosis were established using AlCl_3_, and the effects of DDT were evaluated. We found that DDT treatment for 48 h significantly increased cell viability and reduced the release of lactate dehydrogenase (LDH) in AlCl_3_-induced PC12 cells. Moreover, DDT attenuated AlCl_3_-induced oxidative stress damage by increasing antioxidant activities and apoptosis through mitochondrial apoptotic pathways. Additionally, DDT treatment significantly activated the Sirtuin 1 (SIRT1) -mediated Akt/nuclear factor E2 related factor 2 (Nrf2)/heme oxygenase-1 (HO-1) pathways to limit AlCl_3_-mediated neurotoxicity. Our data indicated that DDT potently inhibited AlCl_3_-induced oxidative-stress damage and apoptosis in neural cells by activating the SIRT1-mediated Akt/Nrf2/HO-1 pathway, which provides further support for the beneficial effects of DDT on Al-induced neurotoxicity.

## Introduction

Neurological disorders have aroused significant concern among health scientists globally because diseases such as Alzheimer’s, Parkinson’s, and dementia can all cause lifelong disability (Singh et al., 2018). Aluminum (Al) is considered to be a pathological factor for various neurological and neurodegenerative diseases and can cause nerve damage (Rizvi et al., 2014; Nampoothiri et al., 2015). The neurotoxicity of aluminum can cause oxidative brain damage, trigger apoptosis, and ultimately, irreversibly damage neurons (Myhre and Utkilen, 2013; Prakash, 2013).

Oxidative stress and mitochondrial apoptosis have long been implicated in a variety of neurodegenerative disorders, such as Alzheimer’s disease (AD), Parkinson’s disease (PD), and stroke (Zhang J. et al., 2017; Alberdi et al., 2018; Dai et al., 2018). The mechanism underlying aluminum neurotoxicity is poorly understood, but damage to the mitochondria and production of ROS are considered key contributors to aluminuminduced neurodegenerative disease (Leuner et al., 2007). Mitochondria are particularly susceptible to ROS-induced damage (Wei and Lee, 2002). Increased mitochondrial oxidative damage has also been observed in early pathological events leading to neurodegeneration (Beal, 1996). Aluminum-induced enhanced ROS production has been shown to cause DNA damage and finally apoptotic cell death (Alolayan et al., 2015). Protecting mitochondria from oxidative damage should be an effective therapeutic strategy against aluminum-induced oxidative stress and ensuing apoptosis.

Sirtuin 1 (SIRT1) plays protective roles against several neurodegenerative diseases including Alzheimer’s, Parkinson’s, and motor neuron diseases (Guarente, 1999). Many studies have indicated that brain derived neurotrophic factor (BDNF) expression, Akt phosphorylation, and nuclear factor E2 related factor 2 (Nrf2) acetylation/deacetylation are mediated and critically controlled by SIRT1 (Pillai Vinodkumar et al., 2014; Ma et al., 2018). Moreover, the phosphatidylinositol 3-kinase (PI3K)/Akt signaling pathway plays a critical role in apoptosis (Zhu et al., 2016), which is important in maintaining mitochondrial integrity *via* the phosphorylation of proteins. Recent studies have demonstrated that pathological ROS accumulation alters the activity of the PI3K/Akt signaling pathway, which then leads to oxidative stress damage and apoptosis (Guan et al., 2014). In addition, Nrf2, a key redox-regulated gene, has a critical role in alleviating oxidative stress, and the level of Nrf2 in the nuclei is decreased in patients with neurological disorders (Niedzielska et al., 2016). Recently, accumulating studies have reported that activated nuclear Nrf2 further regulates several endogenous redox-regulated enzymes, such as heme oxygenase-1 (HO-1) *via* the PI3K/Akt pathways (Zhu et al., 2018).

DiDang Tang (DDT) is a traditional Chinese medical decoction. It contains two plants used in traditional Chinese medicine, rhubarb (*Rheum palmatum L*.), and peach seed (*Prunus persica L. Batsch*), and two animals used in traditional Chinese medicine, leeches (*Whitmania pigra Whitman*) and gadflies (*Tabanus mandarinus Schiner*). The Treatise on Cold Pathogenic Diseases pointed out that DDT has the efficacy to improve viscera and removes blood stasis, and it has been widely used to treat neurodegeneration and stroke in nowadays (Chen, 2012; Xu et al., 2015a; Xu et al., 2015b; Xu et al., 2016). In addition, previous clinical studies showed that promoting blood circulation for removing blood stasis therapy could effectively treat cerebral hemorrhage (Ma et al., 2011; Wang et al., 2013; Li et al., 2015). Our previous *in vivo* study showed that DDT has neuroprotective effects, which can increase the expression of BDNF, tyrosine kinase B, and vascular endothelial growth factor (Ren et al., 2013). Studies have shown that the therapeutic effect of DDT is related to antioxidants, and it could effectively improve plasma superoxide dismutase (SOD) activity and decrease plasma malondialdehyde (MDA) content in PC12 cells (Huang et al., 2002; Huang et al., 2018). Previous reports have also shown that DDT can up-regulate B-cell lymphoma-2 (Bcl-2) mRNA and down-regulate Bax mRNA expression in hippocampal neurons in Aβ-induced AD cell model (Li et al., 2014) and the main component of DDT, rhubarb, has anti-oxidative and anti-apoptotic effects, which protect the nervous system in rats with ischemic stroke (Lin et al., 2018).

However, the effect and molecular mechanisms underlying the neuroprotective effects of DDT upon exposure to Al-induced oxidative stress and apoptosis remain elusive. Here, we investigate the protective effect of DDT on oxidative stress and mitochondrial apoptosis and establish the molecular mechanism of DDT by regulating the SIRT1-mediated Akt/Nrf2/HO-1 pathway in an AlCl_3_-induced neurotoxicity model of PC12 cells. Our study could provide a new insight on the protective effect of DDT against Al-induces neurotoxicity.

## Materials and Methods

### Reagents

Aluminum chloride (AlCl_3_.6H_2_O) was purchased from Sigma-Aldrich (St. Louis, MO, USA). Cleaved Caspase-3 (17, 19 kDa, #9664), PARP (116, 89 kDa, #9532), Bcl-2 (26 kDa, #3498), Bax (23 kDa, #2772), Akt (60 kDa, #9272), p-Akt (Ser 473, 60 kDa, #4060), HO-1 (28 kDa, #43966), SIRT1 (120 kDa, #9475), and GAPDH (37 kDa, #5174) antibodies were obtained from Cell Signaling Technology (Beverly, MA, USA). Nrf2 (110 kDa, ab137550) antibody was obtained from Abcam (Cambridge, MA, US). LY294002 was from Selleck Chemicals (S1105, Houston, TX, US). Test kits of the superoxide-dismutase (SOD, #A001-1), catalase (CAT, #A007), glutathione-peroxidase (GSH-Px, #A005), and malonaldehyde (MDA, #A003-1) detection kits were purchased from Nanjing Jiancheng Bioengineering Institute (Nanjing, China). Reactive oxygen species (ROS) assay kits were purchased from Beyotime Biotechnology (Shanghai, China). Annexin V-FITC/PI kit was purchased from BD Biosciences (San Jose, CA, US). iScript cDNA synthesis kit and SYBR Green master mixture were obtained from Bio-Rad (Hercules, CA, US).

### Preparation of DiDang Tang Extract

Material samples from DDT were purchased from Beijing General Pharmaceutical Corporation (Beijing, China) and kindly provided by the Department of Pharmacy at the Affiliated Hospital of Changchun University of Traditional Chinese Medicine. The DDT contained four components, including rhubarb (Chinese name: Dahuang, Latin name: *Rheum palmatum L*., Family: Polygonaceae, Batch number: 170916, Part used: root and rhizome), leeches (Chinese name: Shuizhi, Latin name: *Whitmania pigra Whitman*, Family: Hirudinidae, Batch number: 170824, Part used: whole animal), peach seed (Chinese name: Taoren, Latin name: *Prunus persica L. Batsch*, Family: Rosaceae, Batch number: 171011, Part used: seed), and gadflies (Chinese name: Mengchong, Latin name: *Tabanus mandarinus Schiner*, Family: Tabanidae, Batch number: 171015, Part used: whole animal) with a weight ratio of 5:3:10:3. The aqueous extract of DDT was prepared and stored at -80°C as previously described (Huang et al., 2018). The yield of aqueous extract of DDT was 22.3%.

**Figure 1:**
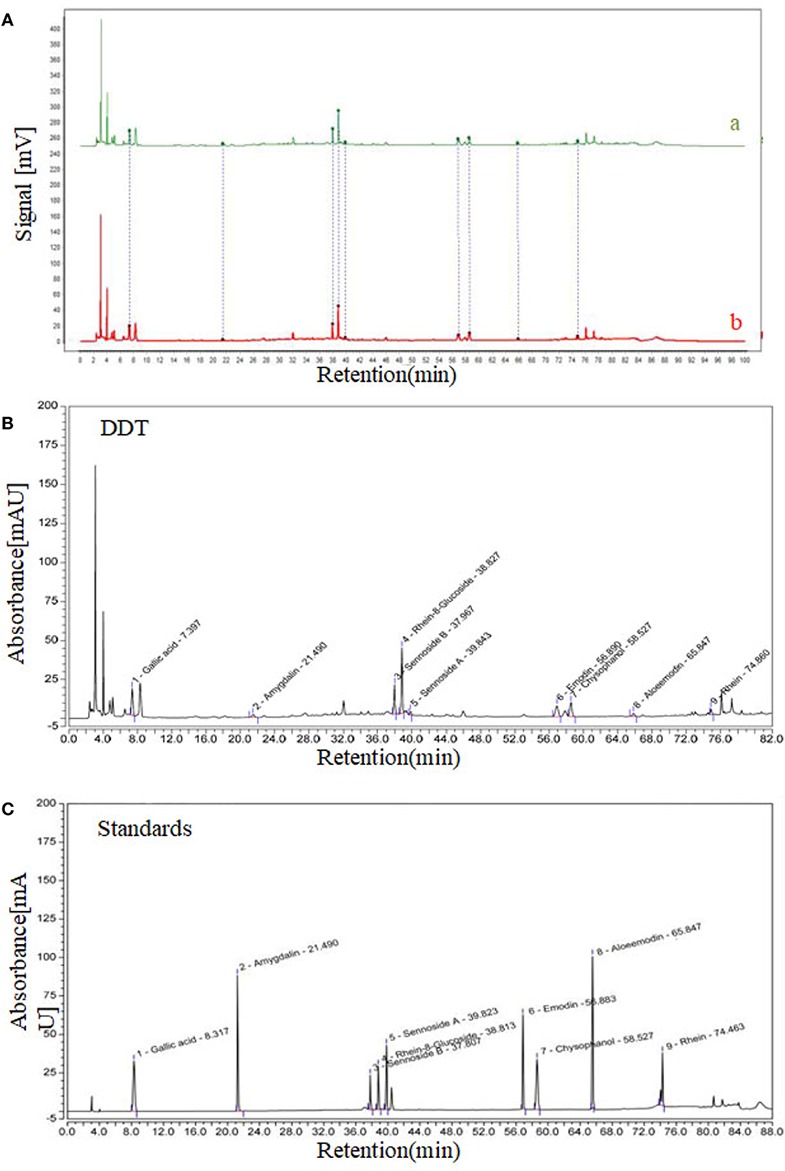
High-performance liquid chromatography (HPLC) chromatogram of DiDang Tang (DDT). **(A)** HPLC chromatograms of DDT (b) and the control fingerprint chromatogram (a) using National Pharmacopeia Committee Chinese Medicine Fingerprint Similarity Evaluation System (2004A) Software are shown. **(B)** DDT levels were determined using HPLC. **(C)** Gallic acid, amygdalin, sennoside B, rhein-8-glucoside, sennoside A, emodin, chrysophanol, aloeemodin, and rhein were identified by HPLC.

As we reported, we have established a method for the detection of active ingredients from DDT *via* high-performance liquid chromatography (HPLC, Agilent, Santa Clara, CA, US) (Huang et al., 2018). As shown in **Figure 1A**, our HPLC chromatogram of DDT is basically consistent with Huang’s experimental results. Eighteen major peaks of DDT extract were identified using HPLC (**Figure 1B**). Gallic acid, amygdalin, sennoside B, rhein-8-glucoside, sennoside A, emodin, chrysophanol, aloe-emodin, and rhein in DDT were identified by comparing the retention time from high-performance liquid chromatography (**Figure 1C**) with good reproducibility.

### Cell Culture and AlCl_3_-Induced·Injury Modeling

The PC12 cell line was obtained from the American Type Culture Collection (Manassas, VA, US). Cells were cultured in RPMI-1640 medium, with 5% fetal bovine serum (FBS, CLARK Bioscience, Claymont, DE, US), 10% horse serum (CLARK Bioscience), 100 U/ml penicillin (Biosharp, Hefei, China), and 100 μg/ml streptomycin (Biosharp) at 37°C in a humidified atmosphere with 5% CO_2_. PC12 cells were seeded in 96-well plates at a density of 3 × 10^3^ cells per well for 24 h and incubated with nerve growth factor (NGF) (50 ng/ml, Gibco, New York, NY, US) for another 72 h. After cell differentiation, media were replaced with media containing AlCl_3_ (1, 2, 4, 5, 6, 8, and 10 mM) or DDT (3.125, 6.25, 12.5, 25, 50, 100, and 200 μg/ml) for 48 h.

### Cell Viability Assay

Differentiated PC12 cells were seeded in 96-well plates at a density of 3 × 10^3^ cells/well in 100 μl of medium. After DDT treatment, AlCl_3_ incubation, or both, cell viability was assessed using 3-(4,5-dimethylthiazol-2-yl)-2,5-diphenyltetrazolium bromide (MTT) assay. Ten microliters of MTT (Sigma-Aldrich, St. Louis, MO, US) assay solution (5 mg/ml) was added to each well and the plates were further incubated for 4 h at 37°C. Medium containing MTT was removed and precipitants were solubilized in DMSO (150 μl/well). Absorbance was measured at 490 nm using a microplate reader (TECAN Infinite M200pro, ZH, Switzerland). All measurements were performed in triplicate.

### Flow Cytometry Analysis of ROS and Apoptosis

PC12 cells were treated with different doses of DDT for 48 h, and then incubated with 4 mM of AlCl_3_ for 6 h in the 6-well plates. According to the manufacturer’s instructions, ROS levels from untreated- and DDT-treated cells were detected using the fluorescent probe, dihydroethidium (DHE). In brief, after incubation with different treatments, the cells were washed with PBS and subsequently incubated with 2 μM of DHE at 37°C in the dark for 30 min. A total of 1 × 10^4^ cells per sample were collected and analyzed for ROS production using a flow cytometer (FACS Calibur™, BD Biosciences) (Hu et al., 2019).

For apoptosis, after treatment with DDT and/or AlCl_3_ for 48 h, SIRT1 activator (Resveratrol, Res), SIRT1 inhibitor (Nicotinamide, NAM), and incubation with AlCl_3_, the cells were harvested and incubated in binding buffer containing 6 μl Annexin VFITC and 10 μl PI in the dark for 15 min to determine the percentages of apoptotic cells by flow cytometry (BD Biosciences) (Yin et al., 2018).

### Lactate Dehydrogenase (LDH) Activity

PC12 cells were plated in a 96-well plate (3 × 10^3^ cells/well). After 48 h of DDT treatment, and/or AlCl_3_ incubation, the activity of LDH was measured using an assay kit (Jiancheng Bioengineering Institute), according to the manufacturer’s introduction. The LDH leakage was determined as the ratio of LDH activity in the culture medium to the total LDH activity in the cell lysate and medium at a wavelength of 450 nm (Lara-GuzmÃn et al., 2018).

### Mitochondrial Transmembrane Potential (MMP) Measurement

MMP of PC12 cells was determined using JC-1 fluorescent dye (Beyotime Biotechnology), which could aggregate in mitochondrial matrix and formed Jaggregates with red fluorescence when the MMP was at a high state. In contrast, when MMP was at a low state, JC-1 could not aggregate but existed as a monomer with green fluorescence. According to the manufacturer’s instructions, cells were cultured in 96-well plates, followed by treatment with NGF, and then incubated with AlCl_3_ (4 mM), various concentrations of DDT, and combinations of these. Cells were stained with JC1 probe in the dark at 37°C for 30 min, washed twice with PBS, and observed under a fluorescent microscope (Olympus, Japan) (Dong and Zhang, 2018).

### Western Blot Analysis

Cells were lysed in radioimmunoprecipitation assay (RIPA) buffer, and protein concentration was measured using a bicinchoninic acid (BCA) protein assay kit (Beyotime Biotechnology). Proteins (30 μg) were separated by 10% or 12% SDSPAGE and electro-transferred to polyvinglidene fluoride (PVDF) membranes. After blocking with 5% non-fat milk, the membranes were incubated with specific primary antibodies (1:1000) overnight at 4°C. After incubation with appropriate secondary antibodies (1:5000) for 1 h at room temperature (Ren et al., 2019). After incubation with chemiluminescence substrate, protein bands were visualized using FluorChem Imager System (ProteinSimple, San Jose, CA, US) and densitometric intensity was analyzed quantitatively using AlphaView Software (ProteinSimple). After normalization to internal control for quantitative analysis, the ratio of protein of interest from Al and/or DDT group to that of the control group was considered as the relative expression.

To analyze the effect of DDT on the translocation of Nrf2, cytosolic and nuclear fractions from different groups were separated using nuclear and cytoplasmic protein extraction kits (Bestbio Science, Nanjing, China). After washing with PBS, cells were collected and extracted using cytoplasmic lysis buffer to obtain the supernatants as cytoplasmic fraction. Then, the pellets were added to nuclear lysis buffer and centrifuged at 10,000 rpm to obtain nuclear fraction. Nrf2 expression in cytoplasm and nucleus were analyzed and shown as the ratio of nuclear/cytoplasmic Nrf2 for evaluating Nrf2 translocation.

### Reverse Transcription-Quantitative PCR (RT-qPCR)

Differentiated PC12 cells were seeded at a density of 2 × 105 cells/well in a 6-well plate. The cells were treated with DDT and/or AlCl3 for 48 h for measurement of Bax and Bcl-2 mRNA expression. Total RNA from cultured cells was extracted with TRIzol (Invitrogen, Carlsbad, CA, US). Next, 1 μg of RNA was reverse-transcribed into cDNA by the iScript cDNA Synthesis kit (Bio-Rad). Subsequently, qPCR was performed using a Bio-Rad CFX96 system and set as: 95°C for 5 min, 95°C for 15 s, 60°C for 30 s, and 72°C for 30 s for 40 cycles. The sequences of the real-time PCR primers were as follows: rat Bax forward: 5′- ACACCTGAGCTGACCTTG -3′, reverse: 5′- AGCCCATGATGGTTCTGATC -3′; rat Bcl-2 forward: 5′-CATGCGACCTCTGTTTGA -3′, reverse: 5′- GTTTCATGGTCCATCCTTG -3′; rat GAPDH forward: 5′-GGAGCGAGATCCCT CCAAAAT-3′, reverse: 5′-GGCTGTTGTC ATACTTCTCAT. Relative levels of Bcl-2 and Bax were calculated using the 2^-ΔΔCt^ method after normalization to GAPDH (Choi et al., 2017).

### Biochemical Analysis

Cells from untreated and DDT-treated groups were lysed in RIPA buffer. The activities of SOD and catalase (CAT), and the contents of glutathione peroxidase (GSHPx) and MDA, were determined by assay kits (Jiancheng Bioengineering Institute), according to the manufacturer’s instructions (Zhang L. L. et al., 2017).

### Statistical Analysis

Data from three independent experiments are presented as the mean ± standard deviation and analyzed using GraphPad Prism 6.0 (GraphPad Software, San Diego, CA, US). For multiple comparisons, data were subjected to one-way ANOVA test (Turkey’s *post hoc*) to determine statistical significance. For all statistical tests, *p* < 0.05 was considered statistically significant.

## Results

### AlCl_3_ Decreases Viability and Promotes Apoptosis in PC12 Cells

**Figure 2:**
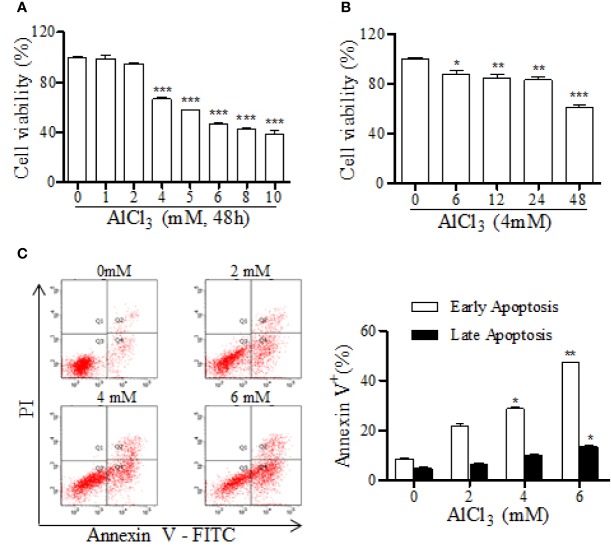
AlCl_3_·decreases cell viability and increases apoptosis in PC12 cells. **(A)** The neurotoxic effect of AlCl_3_ with different concentrations (1, 2, 4, 5, 6, 8, and 10 mM) was determined by an MTT assay. **(B)** The effects of AlCl_3_ incubation at 4 mM for different hours (6, 12, 24, and 48 h) on cell viability were assessed by an MTT assay. **(C)** After 48 h incubation, AlCl_3_-induced apoptosis was determined with annexin-V/PI staining, followed by FACS analysis. The bar graph on the right shows the percentages of cells in early and late apoptosis. Values are means ± standard derivation from three independent experiments. ^*^p < 0.05, ^**^p < 0.01, ^***^p < 0.001 vs. 0 group.

To investigate the effect of DDT on cell viability and apoptosis, we first determined the modeling conditions for AlCl_3_-induced neurotoxicity in PC12 cells. The effects of AlCl_3_ on cell viability and apoptosis were assessed by an MTT assay and flow cytometry. AlCl_3_ at the concentrations of 4, 5, 6, 8, and 10 mM induced decreases of PC12 cell viabilities to 66.6%, 57.4%, 46.5%, 42.8%, and 38.7% the levels in the vehicle group, respectively (**Figure 2A**). Moreover, AlCl_3_ incubation for 6, 12, 24, and 48 h decreased PC12 cell viabilities to 87.7%, 84.8%, 83.2%, and 61.2%, respectively, compared to that of the vehicle group (**Figure 2B**). In addition, we further examined the percentages of apoptotic cells at different concentrations of AlCl_3_ (2, 4, and 6 mM). As shown in **Figure 2C**, AlCl_3_ at 4 mM for 48 h significantly induced the increases of early and late apoptotic cells from 13.2% to 38.6%, compared with that of the vehicle group. Taken together, these results indicated that AlCl_3_ incubation decreased neuronal-like cell viability and induced apoptosis. In this study, we used AlCl_3_ incubation at 4 mM for 48 h to establish a model of apoptosis in PC12 cells.

### DDT Protects PC12 Cells From Damage and Apoptosis Induced by AlCl_3_

**Figure 3:**
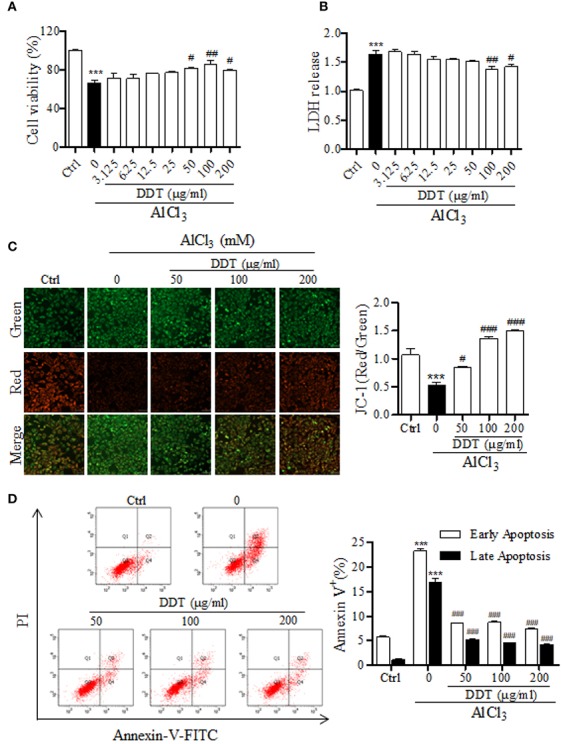
DDT protects PC12 cells from damage and apoptosis induced by AlCl_3_. **(A)** After treatment with DDT and/or AlCl_3_ for 48 h, PC12 cell viability was assessed by an MTT assay. **(B)** LDH release was measured in PC12 cells treated with AlCl_3_, DDT-treated, or both for 48 h using an LDH assay kit. **(C)** Following treatment with DDT and/or AlCl_3_ for 48 h, PC12 cells were incubated with a JC-1 probe to measure mitochondrial membrane potential. The bar graph on the right represents the fluorescent intensity of JC-1. **(D)** Cells treated with DDT, AlCl_3_, or both were stained with Annexin V and PI, and then were analyzed by flow cytometry. Data are expressed as the average percentages of cells in early and late apoptosis. Values are means ± standard derivation from three independent experiments. Ctrl: control group. ^***^p < 0.001 vs. control group; ^#^*p* < 0.05, ^##^*p* < 0.01, ^###^*p* < 0.001 vs. AlCl_3_-induced model group.

To study the protective effect of DDT on neural cells, differentiated PC12 cells were treated with different doses of DDT and exposed to 4 mM of AlCl_3_ for 48 h. As the results showed in **Figure 3A**, DDT increased cell viability at concentrations of 50, 100, and 200 μg/ml. DDT treatment also obviously inhibited the release of LDH at the concentration of 100 and 200 μg/ml (**Figure 3B**). Further, the inner MMP breaks down early during mitochondria-dependent apoptosis (Li X. Q. et al., 2019). To further establish whether the neuroprotective effect of DDT was mediated *via* inhibition of mitochondrial apoptosis, we detected MMP and apoptosis in AlCl_3_-exposed PC12 cells. As shown in **Figure 2C**, AlCl_3_ induced a 0.5-fold reduction in the ratio of red/green fluorescence, compared with that of the control group. DDT significantly increased MMP 1.36-1.50-fold over the AlCl_3_-induced group (**Figure 3C**). In addition, flow cytometry analysis revealed that DDT visibly suppressed the rates of early and late apoptosis induced by AlCl_3_ in a concentration-dependent manner (**Figure 3D**). These results indicated that DDT was able to protect PC12 cells from AlCl_3_-induced apoptosis.

### DDT Inhibits Mitochondrial Apoptosis-Related Signaling Pathways Induced by AlCl_3_ in PC12 Cells

**Figure 4:**
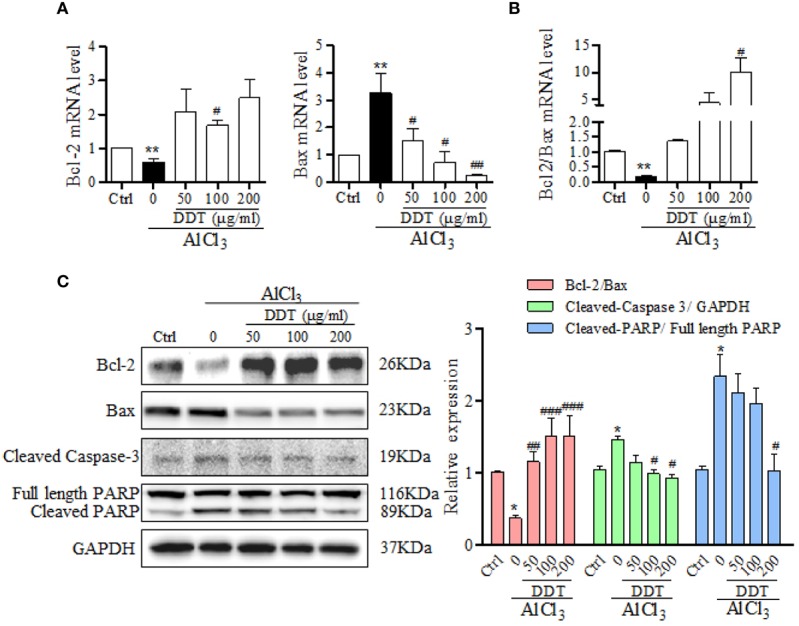
DDT inhibits apoptotic-related signaling pathway induced by AlCl_3_ in PC12 cells. **(A)** The mRNA levels of Bcl-2 and Bax in AlCl_3_-induced PC12 cells treated with different concentrations of DDT for 48 h were detected by qPCR analysis. β-actin was an internal control. **(B)** The ratio of Bcl-2/Bax at the mRNA level is shown. **(C)** The levels of Bcl-2, Bax, cleaved caspase-3, and cleaved PARP in PC12 cells after 48 h of AlCl_3_ incubation and DDT treatment were detected by western blot analysis. The relative expression of each protein was quantified based on normalization to GAPDH level and is shown on the right. Values are means ± standard derivation from three independent experiments. Ctrl: control group. ^*^p < 0.05, ^**^p < 0.001 vs. control group; ^#^*p* < 0.05, ^##^*p* < 0.01 , ###p < 0.001 vs. AlCl_3_-induced model group.

To further analyze the mechanism by which DDT acts against AlCl_3_-induced apoptosis, the levels of apoptosis-related genes and proteins, such as Bcl-2, Bax, caspase-3, and poly-ADP ribose polymerase (PARP), were examined using qPCR and western blot analysis. qPCR analysis revealed that DDT treatment significantly increased the mRNA expression of Bcl-2 and inhibited the mRNA expression of Bax relative to the AlCl_3_-induced group (**Figure 4A**). As shown in **Figures 4B, C**, AlCl_3_ incubation reduced the ratio of Bcl-2/Bax at the mRNA and protein levels, which was significantly reversed by DDT treatment. Western blot analysis showed that the increased levels of cleaved PARP and cleaved caspase-3 in AlCl_3_-induced PC12 cells were antagonized by DDT treatment for 48 h (**Figure 4C**). These findings suggested that DDT inhibited PC12 cell apoptosis by regulating the mitochondrial apoptotic pathways.

### DTT Treatment Reduces AlCl_3_-Induced Oxidative Stress in PC12 Cells

**Figure 5:**
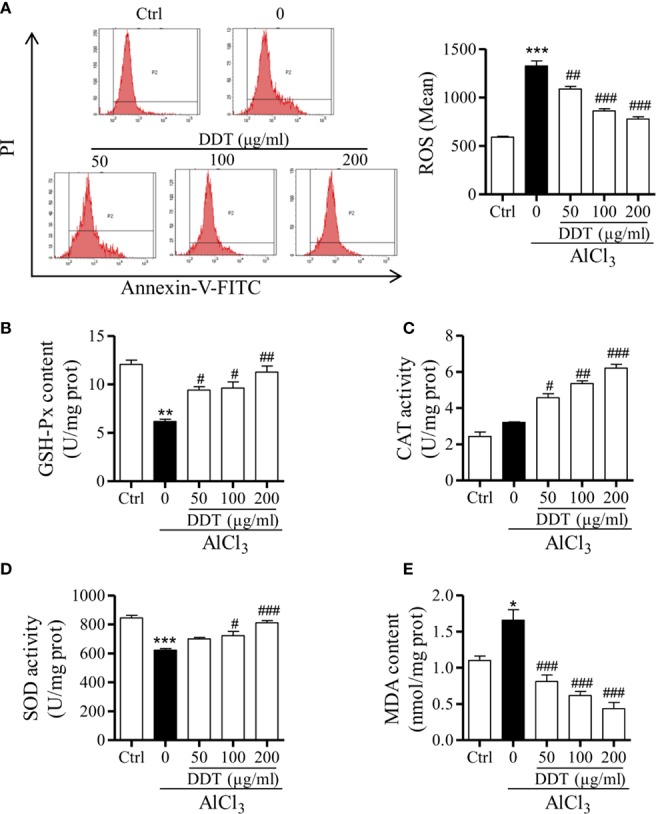
DTT treatment reduces AlCl_3_-induced oxidative stress in PC12 cells. **(A)** Intracellular ROS levels from PC12 cells pretreated with DDT for 48 h and AlCl_3_ induction for 6 h were evaluated by flow cytometry. The bar graph on the right shows the fluorescence intensity of ROS production. **(B–E)** The effects of DDT on GSH-Px content, CAT, and SOD activities, and MDA content were assessed using biochemical assay kits. Values are means ± standard derivation from three independent experiments. Ctrl: control group. ^*^p < 0.05, ^**^p < 0.01, ^***^p < 0.001 vs. control group; ^#^*p* < 0.05, ^##^*p* < 0.01, ^###^*p* < 0.001 vs. AlCl_3_-induced model group.

Based on previous study, we first confirmed AlCl_3_ (4 mM) for 6 h incubation to establish an oxidative stress cell model in PC12 cells by flow cytometry. To evaluate the effect of DDT on oxidative stress, PC12 cells were treated with different doses of DDT for 48 h, and then incubated with 4mM of AlCl_3_ for 6 h. Compared with the control group, AlCl_3_ significantly increased the level of intracellular ROS in PC12 cells, which was decreased by DDT pretreatment for 48 h (**Figure 5A**). Then, the effects of DDT on GSH-Px content, SOD and CAT activities, as well as MDA content were investigated by biochemical analysis. As shown in **Figure 5B**, AlCl_3_ decreased the content of GSH-Px from 12.05 to 6.17 U/g protein, and DDT significantly recovered the decrease of AlCl_3_induced group to 9.40, 9.63, and 11.26 U/g protein. Moreover, the activities of CAT and SOD were visibly decreased by AlCl_3_ incubation relative to the control group (**Figures 5C, D**). Compared with the AlCl_3_-induced group, DDT treatment significantly increased the activity levels of CAT and SOD in response to AlCl_3_ around 2.0-fold and 1.3-fold, respectively (**Figures 5C, D**). AlCl_3_ induction also increased the content of MDA in PC12 cells, which was effectively antagonized by DDT treatment in a concentration-dependent manner (**Figure 5E**). Taken together, these results suggested that DDT decreased ROS production and improved the antioxidant capacity induced by AlCl_3_ in PC12 cells.

### DDT Protects PC12 Cells Against AlCl_3_-Induced Oxidative Stress and Apoptosis by Activating the SIRT1-Mediated Akt/Nrf2/HO-1 Pathway

**Figure 6:**
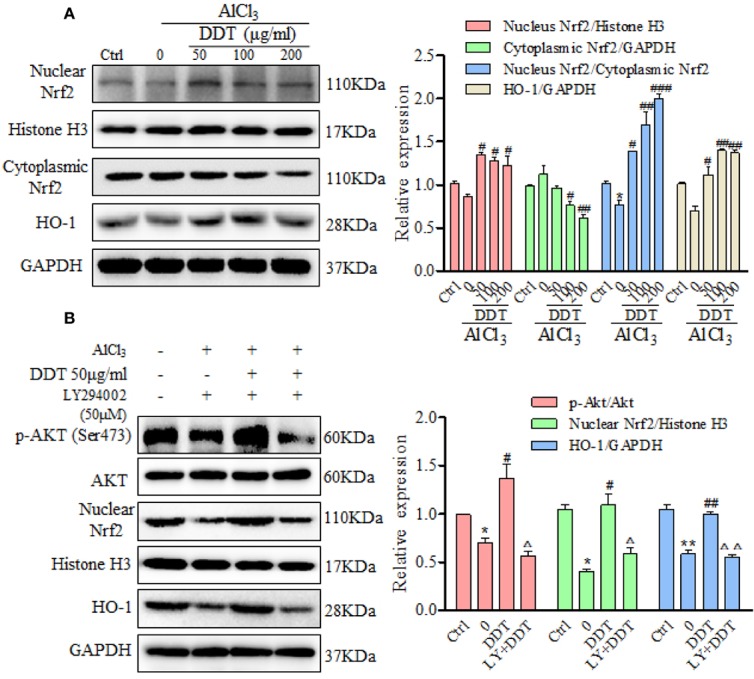
DDT protects PC12 cells against AlCl_3_ by activating the Akt/Nrf2/HO1 pathway. **(A)** After the pretreated with DDT for 48 h and AlCl_3_ induction for 6 h, the levels of nuclear and cytoplasmic Nrf2, and HO-1 in PC12 cells were detected by western blot analysis. GAPDH and Histone H3 served as loading controls for the cytoplasmic and nuclear fractions, respectively. After normalization to internal loading control, densitometric intensity of each protein was shown as the respective expression. The ratio of relative nuclear Nrf2/cytoplasmic Nrf2 expression also was calculated and is shown on the right. **(B)** PC12 cells were pretreated with the PI3K inhibitor, LY294002 (50 μM) for 8 h, and then treated with AlCl_3_ and/or DDT for 4 h. Phosphorylation of Akt (Ser473), Akt, nuclear Nrf2, and HO-1 were evaluated by western blot analysis. GAPDH was used as a loading control. Values are means ± standard derivation from three independent experiments. Ctrl: control group. ^*^p < 0.05, ^**^p < 0.01 vs. control group; ^#^*p* < 0.05, ^##^*p* < 0.01, ^###^*p* < 0.001 vs. AlCl_3_-induced model group; ^Δ^*p* < 0.05, ^ΔΔ^*p* < 0.01 vs. DDT at 50 μg/ml group. PI3K, phosphatidylinositol 3-kinase; Nrf2, nuclear factor E2 related factor 2; HO-1, heme oxygenase-1.

Several studies reported that Akt/Nrf2 pathway and its target anti-oxidant genes, such as HO-1, have critical roles in the mitigation of oxidative stress and apoptosis (Kumar et al., 2014; Xu et al., 2015a; Xu et al., 2015b), which participated in neurological disorders. Western blot results revealed that AlCl_3_ visibly decreased the expression of nuclear Nrf2 (**Figure 6A**). Treatment with DDT significantly increased nuclear Nrf2 expression and decreased cytoplasmic Nrf2 expression in AlCl_3_-induced PC12 cells (**Figure 6A**). Moreover, the ratio of relative nuclear Nrf2 to cytoplasmic Nrf2 at the protein level was significantly reversed by DDT treatment (**Figure 6A**). Consistently, AlCl_3_ reduced the level of HO-1, which was significantly reversed by DDT treatment (**Figure 6A**). These results suggested that DDT promoted the translocation of Nrf2 from the cytosol to the nucleus and HO-1 activation to protect AlCl_3_-induced PC12 cell neurological disorders.

The mutual synergy between the PI3K/Akt and Nrf2 signaling pathways can control the cell defense system and so mitigate oxidative damage and apoptosis (Cui et al., 2018). In this study, we used LY294002, a PI3K-specific inhibitor, to further investigate whether PI3K/Akt signaling mediates the upregulation of Nrf2 and HO-1 by DDT treatment in the AlCl_3_-induced PC12 cells. We found that AlCl_3_ incubation reduced the phosphorylation of Akt at Ser473, which was reversed by DDT at 50 μg/ml (**Figure 6B**). LY294002 (50 μM) treatment returned the DDT-enhanced p-Akt level in the AlCl_3_-induced PC12 cells (**Figure 6B**). DDT treatment also increased the levels of nuclear Nrf2 and HO-1 over those of the AlCl_3_-treated group, but this effect was blocked by LY294002 (**Figure 6B**). These results indicated that DDT protected PC12 cells from AlCl_3_-induced oxidative stress damage and apoptosis by activating the Akt/Nrf2/HO-1 signaling pathway.

**Figure 7:**
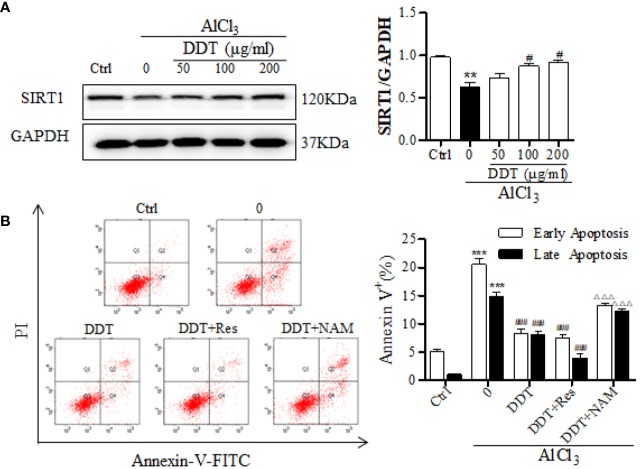
DDT protects PC12 cell apoptosis from AlCl_3_ by activating SIRT1. **(A)** After DDT treatment and AlCl_3_ incubation for 48 h, the level of SIRT1 in PC12 cells was detected by western blot analysis. The intensity was normalized to the intensity of GAPDH and shown on the right. **(B)** PC12 cells were incubated with DDT and AlCl_3_ for 48 h, and/or SIRT1 activator/inhibitor (resveratrol, Res, 10 μM for 24 h; nicotinamide, NAM, 10 mM for 16 h), and then collected to analyze the percentages of early and late apoptosis by flow cytometry analysis. Values are means ± standard derivation from three independent experiments. Ctrl: control group. ^**^p < 0.01, ^***^p < 0.001 vs. control group; ^#^*p* < 0.05, ^###^*p* < 0.001 vs. AlCl_3_-induced model group; ^ΔΔΔ^*p* < 0.001 vs. DDT at 50 μg/ml group. SIRT1, Sirtuin 1.

It has been reported that BDNF expression, Akt phosphorylation, and Nrf2 acetylation/deacetylation are mediated and critically controlled by SIRT1 (Pillai Vinodkumar et al., 2014; Ma et al., 2018). Based on these findings, we analyzed the effect of DDT on the expression of SIRT1 using western blot analysis. The results indicated that AlCl_3_ visibly decreased the expression of SIRT1, which was significantly increased by DDT treatment (**Figure 7A**). SIRT1 inhibitor/activator was used to further analyze the protective mechanism of DDT against AlCl_3_-induced apoptosis through the activation of SIRT1. As shown in **Figure 7B**, flow cytometry analysis showed that AlCl_3_ significantly induced the increases of early and late apoptotic cells from 6.7% to 33.7%, which was visibly suppressed by DDT (16.6% of apoptotic cells). Moreover, DDT with Res led to 12% of the rates of early and late apoptosis, which has a better effect on apoptosis than DDT. However, the combination of DDT with NAM had less protective effect on AlCl_3_ induced PC12 cell apoptosis than DDT does. Taken together, these results indicated that DDT protected neural cells from AlCl_3_-induced oxidative stress damage and apoptosis by activating the SIRT1-mediated Akt/Nrf2/HO-1 signaling pathway.

## Discussion

The neurotoxicity of aluminum can cause oxidative brain damage, further triggering apoptosis, which ultimately causes irreversible damage to neurons (Kawahara and Katonegishi, 2011; Bakour et al., 2017). Substantial efforts have made to identify natural anti-oxidants and anti-apoptotic agents with neuroprotective potential. The present study demonstrates that DDT protected the AlCl_3_-induced PC12 neurotoxicity, which was characterized by an increase in cell viability and in the percentage of apoptotic cells and the cleavages of apoptotic related proteins. Further, DDT inhibited ROS generation and improved antioxidant capacity induced by AlCl_3_. In addition, DDT treatment increased the expression of SIRT1, Akt phosphorylation and upregulated Nrf2 and HO-1 levels. Taken together, these findings support our hypothesis that DDT protects neurons from AlCl_3_-induced oxidative stress and mitochondrial-mediated cell apoptosis through the activation of the SIRT1-mediated Akt/Nrf2/HO-1 pathway.

Studies have shown that Al induces apoptosis in neurons and brain tissues (Savory et al., 2003; Johnson and Kim SHSharma, 2005; Dewitt et al., 2006). SH-SY5Y cells treated with relatively high concentrations of Al caused a decrease in cell viability and apoptosis (Shamasundar et al., 2006). Moreover, AlCl_3_ administered to Kunming mice remarkably increased brain and plasma Al contents, increased lipid peroxidation, and induced hippocampal neuronal damage (Li et al., 2017). Therefore, AlCl_3_ is advantageous for use in the investigation of Al neurotoxicity. In our present study, DDT increased cell viability, reduced the release of LDH, and decreased apoptosis induced by AlCl_3_, which indicated that DDT could decrease neurotoxicity. In order to further confirm the effect of DDT on apoptosis, we measured the expression of Bcl-2, Bax, and cleaved caspase 3 and PARP-1-a protein downstream to caspase-3, which is cleaved by caspases including caspase-3-during apoptotic cell death (Park et al., 2017). DDT treatment down-regulated Bax, up-regulated Bcl-2 expression, inhibited Bax translocation to mitochondria, restrained mitochondria-mediated apoptotic pathways, inhibited the cleavage of caspase-3 and PARP, and inhibited the fragmentation of internucleosomal DNA to protect PC12 cells from apoptosis.

In previous studies, the mechanism of Al toxicity has been poorly understood, but the literature has suggested that Al generates ROS that cause lipid peroxidation, oxidative damage to proteins and DNA, as well as decreased intracellular glutathione (González et al., 2009). As oxidative stress is an important factor in neuronal injury, we re-explored the conditions of oxidative-stress production and found that ROS production was increased most obviously at 6 h of Al exposure. Due to the short modeling time, we chose DDT pretreatment for 48 h and then exposed cells to Al (4 mM, 6 h) to study the effect of DDT on oxidative stress. To further elucidate the neuroprotective potential of DDT and its molecular mechanisms, we evaluated oxidative-stress indices, including the content of GSH-Px and MDA and the activities of SOD and CAT-in response to AlCl_3_ exposure and/or DDT pretreatment. DDT treatment exhibited a significant enhancement of anti-oxidation, which is reflected in decreases ROS production, improves the activity of antioxidant enzymes, and increases the content of anti-oxidants induced by AlCl_3_ in PC12 cells.

The phosphatidylinositol-3-kinase (PI3K)/Akt signaling pathway is a key regulator of cell survival and proliferation that is widely expressed in the central nervous system (Guo et al., 2017). This pathway is also associated with the regulation of various cellular metabolisms involved in neurocyte nutrition and the processes underlying learning and memory (Horwood et al., 2006; Aberg et al., 2008; Chiang et al., 2010). Numerous studies have shown that the PI3K/Akt cascade is inhibited in neurodegeneration and that its activation aids in inhibiting neuronal apoptosis and promoting neuronal survival (Chung et al., 2007; Xue et al., 2011). Studies have shown that SIRT1-dependent deacetylation promotes phosphorylation and activation of Akt by the upstream kinases (Pillai Vinodkumar et al., 2014). Western blot analysis indicated that DDT increased the expression of SIRT1. In addition, DDT treatment promoted Akt phosphorylation to a striking extent, but it was visibly suppressed by LY294002. These results indicated that PI3K/Akt cascade participates in the protective role of DDT in Alinduced neurotoxicity, consistent with previous reports. Moreover, accumulation of ROS induces oxidative stress that mediates the down-regulation of p-PI3K/p-Akt, which results in Nrf2 translocation from the nucleus to the cytosol and produces a low level of anti-oxidant activity (Nakaso et al., 2003; Qi et al., 2012). Studies have reported translocation of Nrf2 from the cytosol to the nucleus, where it activates anti-oxidative enzymes, such as HO-1, and produces a notable anti-oxidative response to counteract escalated ROS-induced oxidative stress and protect against oxidative stress (Loboda et al., 2016; Xu et al., 2019). The Nrf2/HO-1 signaling pathway is considered as a protective molecular mechanism in several pathological processes, particularly oxidative stress, and this pathway is also involved in several neurodegeneration diseases, including AD and PD (Tufekci et al., 2011; Li Y. P. et al., 2019). Levels of Nrf2 localized to the nucleus and the expression level of HO-1 were both significantly higher in cells treated with DDT, the activation of Nrf2 we observe may in part be mediated *via* Akt, increasing antioxidant defenses (**Figure 6**). Taken together, DDT treatment strikingly promoted SIRT1-mediated PI3K/Akt/Nrf2/HO-1 pathway activation in AlCl_3_-induced PC12 cells. However, the expression of p-Akt, Nrf2, and HO-1 were suppressed by LY294002. These results indicated that the SIRT1-mediated Akt/Nrf2/HO-1 signaling pathway might contribute to the neuroprotective effect of DDT.

**Figure 8:**
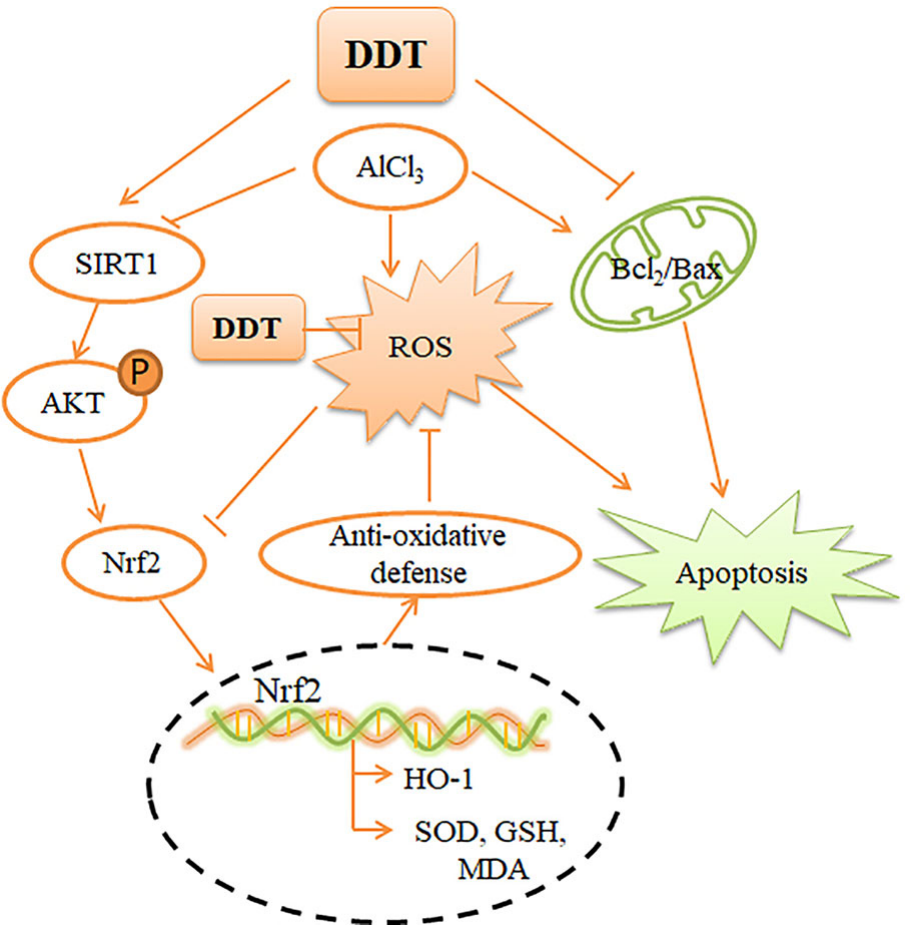
Mechanism by which DDT inhibits oxidative stress and apoptosis in PC12 cells. AlCl_3_-induced intracellular ROS accumulation, which causes intracellular oxidative stress and apoptosis. DDT up-regulates SIRT1-mediated PI3K/Akt signaling, which causes Nrf2 expression and nuclear translocation. Nrf2 activation enhances the downstream, HO-1 expression, which finally protects cells from oxidative stress and apoptosis.

In summary, our results confirmed that ROS and mitochondrial apoptosis were activated in AlCl_3_-induced PC12 cells and were significantly down-regulated in the presence of DDT. In conclusion, our findings indicate that the SIRT1-mediated Akt/Nrf2/HO-1 pathway plays an important role in DDT-regulated protection in PC12 cells (**Figure 8**). Findings from this study provide a theoretical basis for the use of DDT to alleviate aluminum-related neurological diseases.

## Data Availability Statement

All datasets generated for this study are included in the article/supplementary material.

## Author Contributions

JL performed experiments and wrote the manuscript. QH and DMZ prepared and analyzed the drugs. TL, LS, and YZ performed the studies. XT, PX, DXZ, DC and DQZ assisted with the experiments. XL and JW designed the study, analyzed the data, and revised the manuscript.

## Funding

This work was supported by the National Natural Science Foundation of China [Nos. 81473576 and 81774224], the National Key Research and Development Program of China [2018YFC1706002 and 2019YFC1709900], and the Science and Technology Development Plan of Jilin Province [No. 20190101010JH].

## Conflict of Interest

The authors declare that the research was conducted in the absence of any commercial or financial relationships that could be construed as a potential conflict of interest.
